# High fabrication-tolerant narrowband perfect graphene absorber based on guided-mode resonance in distributed Bragg reflector

**DOI:** 10.1038/s41598-019-40945-4

**Published:** 2019-03-12

**Authors:** Sangjun Lee, Hyungjun Heo, Sangin Kim

**Affiliations:** Department of Electrical and Computer Engineering, Ajou University, Suwon, South Korea

## Abstract

We propose the narrowband perfect absorbers with enormously high fabrication tolerance, which consists of a low-contrast grating and a finite distributed Bragg reflector (DBR) layer with an ultrathin absorbing medium (graphene). It is numerically shown that the proposed perfect absorber outperforms the previously proposed schemes in fabrication tolerance. According to the rigorous coupled wave analysis (RCWA) and coupled mode theory (CMT) fitting, over a considerably wide range of grating width and thickness, the proposed absorber provides a proper ratio of leakage rate to loss rate while preserving resonant condition, so that almost perfect absorption (>99.9%) can be obtained. This result is attributed to the strong electric field confinement in the DBR region rather than the grating layer owing to lower index of grating compared to DBR. In addition, without degrading the fabrication tolerance, the bandwidth of the proposed absorber can be controlled by the DBR thickness (the number of pairs) and a narrow absorbing bandwidth of sub-nanometer is achieved with 8.5 Si/SiO_2_ pair stacked DBR.

## Introduction

Due to high conductivity, atomically unltrathin graphene of ~0.34 nm has attracted strong interests in developing high-speed graphene-based photodetectors^1–4^. However, absorption efficiency is ~2.3% for undoped monolayer graphene, so that the absorption should be enhanced greatly for practical high-performance photodetectors. In order to enhance the absorption in the ultrathin absorbing layer, a resonant structure such as a grating or a photonic crystal can be used. Over the past decade, several schemes for perfect absorption in monolayer graphene under one-side illumination have been proposed^5–14^. Most of the perfect absorber schemes are based on a ‘*Single-mode resonator/mirror*’ structure^5,8,9,11–13^ and their resonators are based on the guided-mode resonance (GMR)^15^. In these resonance-based absorbers, their resonant conditions and performances are highly sensitive to the quality of nano-patterned structures such as sidewall slope of a grating, so that precise fabrication is indispensable to high absorption performance. In this work, we propose a novel scheme to achieve graphene-based narrowband perfect absorption with remarkably improved fabrication tolerance which corresponds to several orders of magnitude larger than that of the previously suggested absorbers. Our proposed scheme adopts a grating and a distributed Bragg reflector (DBR), which is also based on the GMR. The DBR functions as a waveguiding region as well as a mirror. Previously, apparently similar schemes, i.e., a GMR-based DBR-adopting perfect absorber schemes were suggested^5,11–13^. In the scheme reported in refs^5,11^, the DBR works as just a mirror and the high-contrast grating (HCG) is mainly responsible for the GMR. In refs^12,13^, the scheme in which both the HCG and the DBR were responsible for the GMR was suggested for an ultranarrow (~0.03 nm) resonance bandwidth. On the other hand, in our proposed scheme, the grating just diffracts the normal incident light into the DBR that works as a main waveguiding region, so that the GMR condition mainly depends on the DBR structure rather than the grating structure. In addition to the improved grating fabrication tolerance, the resonance bandwidth of our proposed *‘DBR-guiding’* mode resonance scheme can be considerably narrow (<1 nm) with a thicker DBR. Resonances via the coupling of the normal incident light into the *‘DBR-guiding’* mode was experimentally demonstrated by Yang *et al*. in the structure comprised of a gold grating on a TiO_2_/SiO_2_ DBR^16^. The authors claimed that the resonant dips in reflection spectra are robust to the environmental changes. However, the application of their *‘DBR-guiding’* mode-based resonance to perfect absorption or the resonance performance dependence on the structural parameters of the gold grating was not considered at all. Besides, if their structure is used to enhance to the absorption of thin layer like the monolayer graphene, the ohmic loss of the gold grating limits a minimum achievable bandwidth of the resonance and a maximum achievable absorption in the thin layer. Another type of DBR-based perfect absorber has also been suggested, in which graphene is located in the high-Q one-dimensional cavity formed by two DBR mirrors^14^. This scheme is based on the strong field confinement in the cavity and required two high-reflection DBRs. So, the total DBR structure is much thicker than that of the GMR-based scheme.

In this work, the fabrication tolerance of our proposed perfect absorber scheme based on the *‘DBR-guiding’* mode resonance has been investigated in comparison to the previously suggested similar schemes. We have also investigated how to improve the fabrication tolerance of the previously suggested scheme by modifying the HCG structure, which reveals that it cannot outperform our proposed scheme. To analyze the performance of the absorbers, we used the rigorous coupled wave analysis (RCWA)^17^. The mechanism of the greatly enhanced fabrication tolerance of our proposed scheme is also analyzed by using the coupled mode theory (CMT)^5,18^.

## Results

### Proposed perfect absorber structure

**Figure 1:**
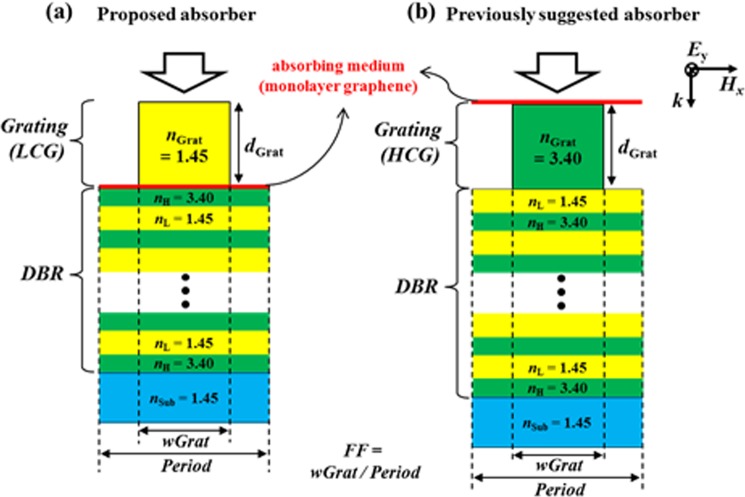
Schematic of (**a**) the proposed perfect absorber with low-index grating (*n*_*Grat*_ = 1.45), and (**b**) the previously suggested perfect absorber with high-index grating (*n*_*Grat*_ = 3.40). For the DBRs of both absorbers, *n*_*H*_ = 3.40, *n*_*L*_ = 1.45, and each optical thickness of the high (Si) or the low (SiO_2_) -index layer in the DBR is set to a quarter of an operating wavelength (1.55 μm) unless otherwise stated. *FF* is defined as the ratio of grating width (*w*_*Grat*_) to *Period*. The red thin layers in (**a**,**b**) indicate monolayer graphene of *t*_*G*_ = 0.34 nm thickness as an absorbing medium.

The proposed absorber (Fig. 1(a)) consists of a one-dimensional (1-D) low-contrast grating (LCG) of SiO_2_, a DBR of finite pairs of Si/SiO_2_ stacked on a glass substrate, and monolayer graphene placed just above the DBR. Refractive indices of SiO_2_, Si, and glass are 1.45, 3.40, and 1.45 respectively. Each optical thickness of the high (Si) or the low (SiO_2_) -index layer in the DBR is set to a quarter of an operating wavelength (1.55 μm). In our DBR structure, the number of Si/SiO_2_ pairs is at least 4.5 to guarantee high reflectivity (*R* > 99.9%)^5^. Figure 1(b) represents the previously suggested perfect graphene absorber structure for comparison^5,11–13^, which is composed of high-contrast gratings (HCG) of Si, monolayer graphene placed just above the HCG, a Si/SiO_2_ DBR on a glass substrate. For fair comparison at the same operation wavelength, the materials and the layer thicknesses of the DBR are modified to be the same as those of our proposed structure, and the operation mechanisms of the previously suggested schemes are preserved. The light of transverse electric (TE) polarization is incident normally from above the grating in both cases.

Although both absorber schemes are based on a ‘*Single-mode resonator/mirror*’ structure^5,8,9,11–13^ and their resonators are based on the GMR^15^, their perfect absorption performance in terms of fabrication tolerance are largely different. As briefly mentioned in the previous section, our proposed scheme (Fig. 1(a)) is based on the *‘DBR-guiding’* mode resonance, that is, the GMR occurs via waveguiding in the DBR region. Whereas, in the previously suggested scheme (Fig. 1(b)), the GMR may be caused solely by the HCG or by both the HCG and the DBR depending on the HCG geometry, which will be discussed later in detail. The difference in the main waveguiding regions of those schemes results in enormous difference in the sensitivities of their absorption performances to the grating geometry.

### Excellent fabrication tolerance of the proposed absorber

For our proposed absorber structure depicted in Fig. 1(a), we designed a perfect absorber by optimizing the grating parameters (*Period*, *d*_*Grat*_, and *w*_*Grat*_ (or *FF*)) to maximize absorption at *λ* = 1.55 μm for a given DBR structure of 4.5 Si/SiO_2_ pairs. The RCWA was used for the absorption calculation and the particle swarm optimization (PSO)^19^ was used in the design. For the permittivity of graphene, Kubo formulation^20,21^ was used with parameters of a graphene thickness of 0.34 nm, Fermi velocity of 10^6^ m/s, and mobility of 0.5 m^2^/Vs. (Supplementary information) Here, undoped graphene was assumed.

**Figure 2:**
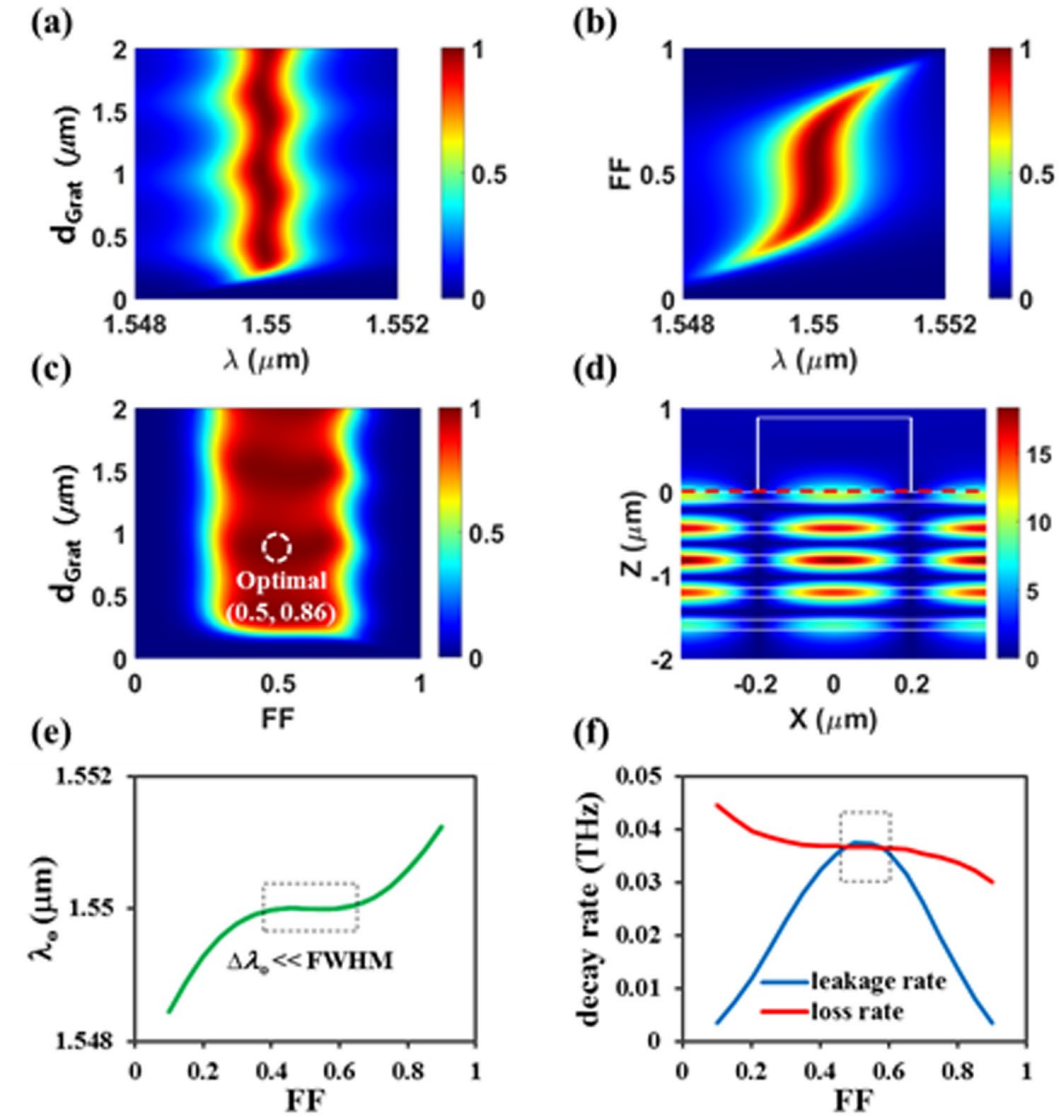
The proposed absorber based on *DBR-guiding* scheme (with optimized *Period* = 0.78344 μm for 4.5 DBR pairs). (**a**) Absorption spectra as a function of *d*_*Grat*_ when *FF* = 0.50. (**b**) Absorption spectra as a function of *FF* when *d*_*Grat*_ = 0.86 μm. (**c**) Absorption map as a function of (*FF*, *d*_*Grat*_) at *λ* = 1.55 μm, and (**d**) electric field distributions (|*E*_*y*_|) at perfect absorption condition (*A* > 99.9% at *FF* = 0.50, *d*_*Grat*_ = 0.86 μm). (**e**) Resonant wavelength and (**f**) two decay rates as a function of *FF*, which are obtained by applying CMT model to (**b**).

Our optimization achieved an absorption larger than 99.9% for *Period* = 0.78344 μm, *d*_*Grat*_ = 0.86 μm, *FF* = 0.50. The optimal period is supposed to satisfy the phase matching condition of the GMR, 2π/*Period* = *n*_eff_ x 2π/*λ*, where *n*_eff_ is the effective refractive index of the guided mode. Since most of the incident light stays in the DBR region in our proposed absorber at resonance (Fig. 2(d)), *n*_eff_ is almost insensitive to the grating geometry variation. Therefore, as long as the period is fixed, the GMR condition and high absorption are sustained over a wide range variation of *d*_*Grat*_ and *FF* in the vicinity of their optimal values. Figure 2(a) shows absorption spectra for the variation of *d*_*Grat*_ with the optimal *Period* and *FF*. The peak absorption wavelength remains in the vicinity of the target resonance wavelength of *λ* = 1.55 μm over a considerably wide range of *d*_*Grat*_. The FWHM of ~1.2 nm is also maintained. The absorption spectrum dependence on *FF* with the optimal *Period* and *d*_*Grat*_ is shown in Fig. 2(b), where we can see that the absorption spectrum almost does not change over a wide range of *FF* around its optimal value of 0.5. To further confirm the robust performance of our designed perfect absorber, in Fig. 2(c), the absorption value at the target resonance wavelength of 1.55 μm is plotted as a function of *d*_*Grat*_ and *FF* with the optimal *Period*. From the optimal point (*FF* = 0.50, *d*_*Grat*_ = 0.86 μm), extremely high absorption value (A > 99.9%) is maintained for 0.44 < *FF* < 0.60 (∆*FF* < 0.16) and 0.843 μm < *d*_*Grat*_ < 0.871 μm (∆*d*_*Grat*_ < 28 nm), while A > 99% for 0.39 < *FF* < 0.64 (∆*FF* < 0.25) and 0.765 μm < *d*_*Grat*_ < 0.945 μm (∆*d*_*Grat*_ < 180 nm). This excellent fabrication tolerance corresponds to several orders of magnitude larger compared to that of the previously suggest structure that will be discussed later.

In addition to the GMR condition sustained over the wide variation of the grating geometry, the feature that the light is confined mostly in the DBR region (Fig. 2(d)) also makes the loss rate experienced by the incident light while staying in the absorber almost constant even if the grating geometry changes. This is an important property because perfect absorption requires the critical coupling condition, that is, balance between an internal decay (loss) rate and an external decay (leakage) rate at the resonance^5,18^. The leakage rate behavior is not easy to assess from the field profile and shows rather strong dependence on the grating geometry in general. To understand the robust absorption performance of our designed device in terms of the critical coupling condition, we analyzed the absorption spectrum characteristics using the CMT.

The proposed absorber can be regarded as one-port resonant system with loss because the DBR works as a reflecting mirror as well as a waveguiding region. According to CMT, absorption efficiency in a lossy one-port resonant system is given by

1 $$A(\omega )=\frac{4{\gamma }_{leak}{\gamma }_{loss}}{{(\omega -{\omega }_{o})}^{2}+{({\gamma }_{leak}+{\gamma }_{loss})}^{2}},$$

where *ω*_o_, *γ*_leak_, and *γ*_loss_ are a resonant frequency, a leakage rate, and a loss rate, respectively^5,18^. The critical coupling condition (*γ*_leak_ = *γ*_loss_) is required to obtain perfect absorption (A(*ω*_o_) = 1).

For the absorption spectra variation when *FF* changes (Fig. 2(b)), we extracted the parameters in (1), that is, *ω*_*o*_, *γ*_leak_ and *γ*_loss_ by fitting CMT model to the absorption spectrum calculated with the RCWA. We confirmed the excellent agreement between the absorption spectra from the RCWA and the CMT model, indicating that the proposed absorber behaves like a lossy one-port resonant system. The extracted fitting parameters for different *FF* are plotted in Fig. 2(e,f). For ~0.39 < *FF* < ~0.64, the resonant wavelength (*λ*_o_) is almost consistent, where the variation of *λ*_o_ is much smaller than the FWHM. Overall, the loss rate shows weak dependence on *FF* as expected, while relatively stronger dependence on *FF* is observed for the leakage rate. However, the slopes of the loss and the leakage rates and their difference are small near the optimal *FF* of 0.5. Near the resonance (*ω* ≈* ω*_o_), the CMT model can be approximated as

2 $$A(\omega )\approx \frac{4({\gamma }_{leak}/{\gamma }_{loss})}{{({\gamma }_{leak}/{\gamma }_{loss}+1)}^{2}},$$

which says almost perfect absorption (A > 99.9%) requires the ratio of the two decay rates should stay in a range of ~0.94 < *γ*_leak_/*γ*_loss_ < ~1.06. The range of *FF* satisfying this condition is indicated by the dotted box (0.44 < *FF* < 0.60) in Fig. 2(f). In our absorber design, *FF* = 0.5 may not be the optimal point in term of the critical coupling condition. However, in terms of the fabrication tolerance, it is the optimal point since both the rates show zero slopes at *FF* = 0.5, where absorption is also high enough (A > 99.9%) to be treated as perfect absorption approximately.

### Fabrication tolerance of the previously suggested absorber with HCG

**Figure 3:**
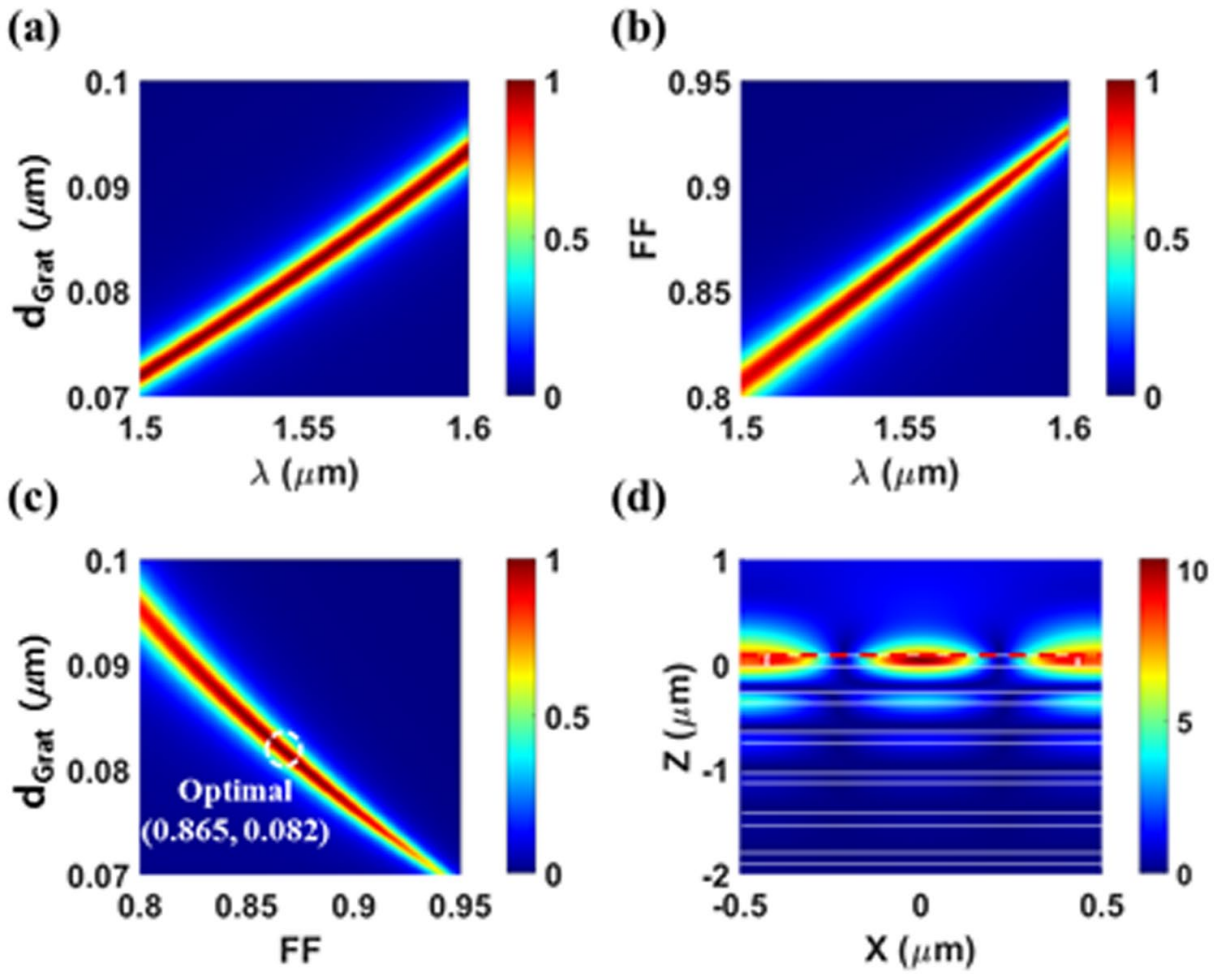
The previously suggested absorber based on *Grating-guiding* scheme (with optimized *Period* = 1 μm for 5 DBR pairs). (**a**) Absorption spectra as a function of *d*_*Grat*_ when *FF* = 0.865. (**b**) Absorption spectra as a function of *FF* when *d*_*Grat*_ = 0.082 μm. (**c**) Absorption map as a function of (*FF*, *d*_*Grat*_) at *λ* = 1.55 μm, and (**d**) electric field distributions (|*E*_*y*_|) at perfect absorption condition (*A* > 99.9%, *FF* = 0.865, *d*_*Grat*_ = 0.082 μm).

**Figure 4:**
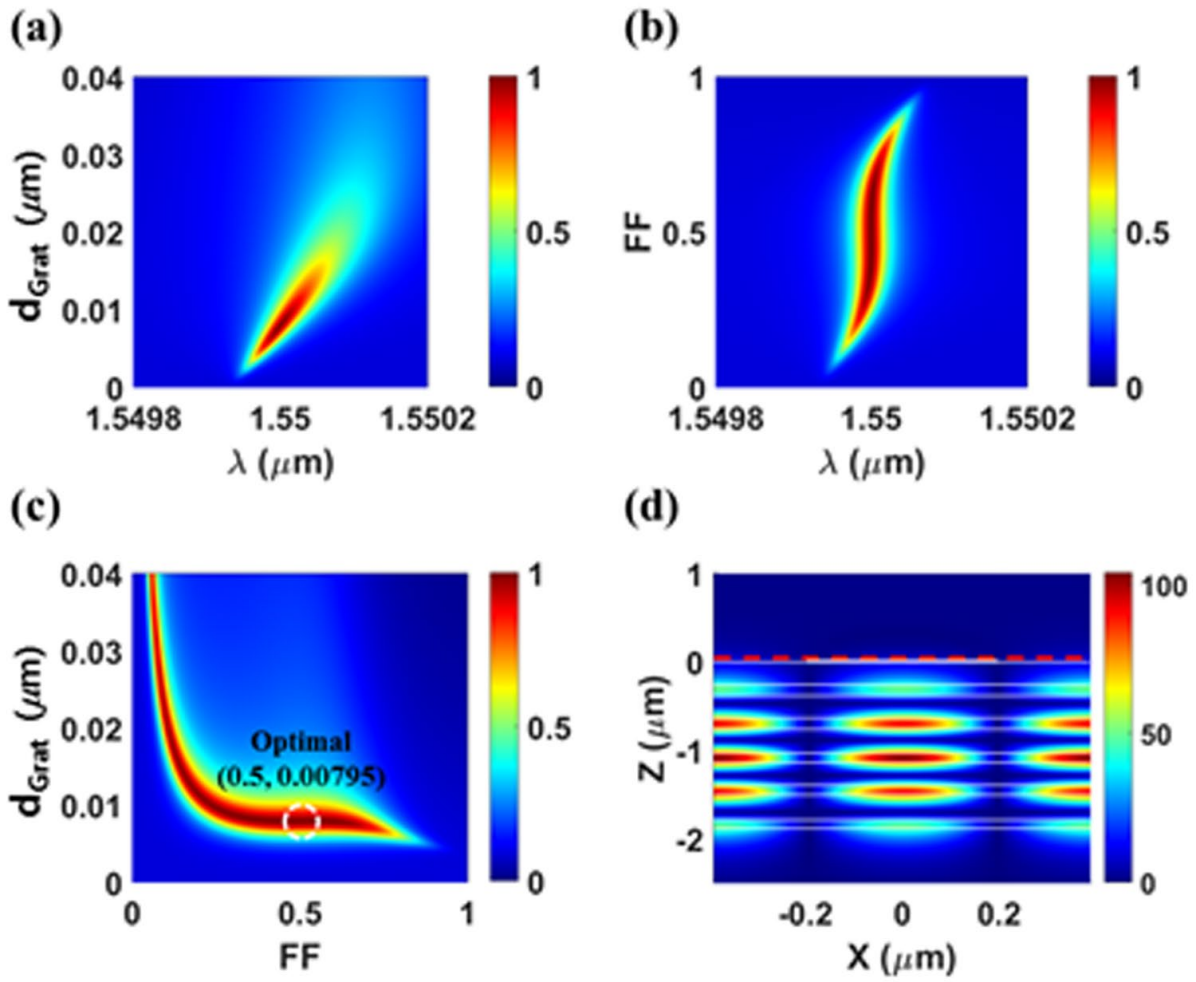
The previously suggested absorber based on *DBR-guiding* scheme (with optimal *Period* = 0.78151μm for 5 DBR pairs). (**a**) Absorption spectra as a function of *d*_*Grat*_ when *FF* = 0.50. (**b**) Absorption spectra as a function of *FF* when *d*_*Grat*_ = 0.00795 μm. (**c**) Absorption map as a function of (*FF*, *d*_*Grat*_) at *λ* = 1.55 μm, and (**d**) electric field distributions (|*E*_*y*_|) at perfect absorption condition (*A* > 99.9% at *FF* = 0.50, *d*_*Grat*_ = 0.00795 μm).

For comparison, we also analyzed the fabrication tolerance of the previously suggested scheme depicted in Fig. 1(b). With a fixed DBR structure of 5 Si/SiO_2_ pairs, we conducted HCG structure optimization to maximize absorption at *λ* = 1.55 μm. Among many possible structures, two representative cases are discussed here: (*Period* = 1 μm, *d*_*Grat*_ = 0.082 μm, and *FF* = 0.865) and (*Period* = 0.78151 μm, *d*_*Grat*_ = 0.00795 μm, and *FF* = 0.5). In both cases, perfect absorption (A > 99.9%) were obtained and their performance dependences on the grating geometry are represented in Figs 3 and 4, respectively. In the first case, the field profile at the resonance (Fig. 3(d)) shows strong field confinement in the HCG region implying the HCG is mainly responsible for the GMR. This case corresponds to the scheme reported in refs^5,11^. As seen in Fig. 3(a,b), the resonance wavelength shows strong dependence on both *d*_*Grat*_ and *FF* as expected. As a result, the absorption value at the target resonance wavelength also shows strong dependence on the grating geometry (Fig. 3(c)). From the optimal point (*FF* = 0.865, *d*_*Grat*_ = 0.082 μm), A > 99% can be obtained only for an extremely narrow parameter range of 0.08189 μm < *d*_*Grat*_ < 0.08215 μm (∆*d*_*Grat*_ < 0.26 nm) and 0.8644 < *FF* < 0.8659 (∆*FF* < 0.0015).

Whereas, in the second case, the HCG is too thin (*d*_*Grat*_ = 0.00795 μm) to support its own guiding mode, so that a large portion of light is confined in the DBR (Fig. 4(d)). This case corresponds the scheme reported in refs^12,13^. As a result of the *‘DBR-guiding’* effect, the tolerance on *FF* is much improved compared to the first case. However, the resonance wavelength and the perfect (or high) absorption feature strongly depend on *d*_*Grat*_ (Fig. 4(a)). As the grating becomes thicker, the graphene layer gets farther away from the guiding region and thus, the loss rate decreases rapidly. This will ruin the critical coupling condition. The resonance wavelength dependence on the HCG thickness seems to be attributed to the Fabry-Perot (*FP*) resonance in the vertical direction due to the large index difference between air and the HCG. This phenomenon is clearer when the HCG is very thick (*d*_*Grat*_ > ~0.25 μm) and strong interaction between the GMRs in the DBR and the FP resonance is observed. (Supplementary information) Consequently, the fabrication tolerance of this case is rather poor as seen in Fig. 4(c). Near the optimal point (*FF* = 0.5, *d*_*Grat*_ = 0.00795 μm), A > 99% can be obtained for a parameter range of 0.00773 μm < *d*_*Grat*_ < 0.00817 μm (∆*d*_*Grat*_ < 0.44 nm) and 0.37 < *FF* < 0.62 (∆*FF* < 0.25). However, one thing to note in this case is that the bandwidth of the absorption spectrum is very narrow (~ 0.04 nm) for the optimal grating geometry as seen in Fig. 4(a,b). This is because of a lower loss rate than that of our design stemming from the location of the graphene. The bandwidth reduction of our design is also possible, which will be discussed below.

### Narrowband absorption by increasing the number of pairs in the DBR

**Figure 5:**
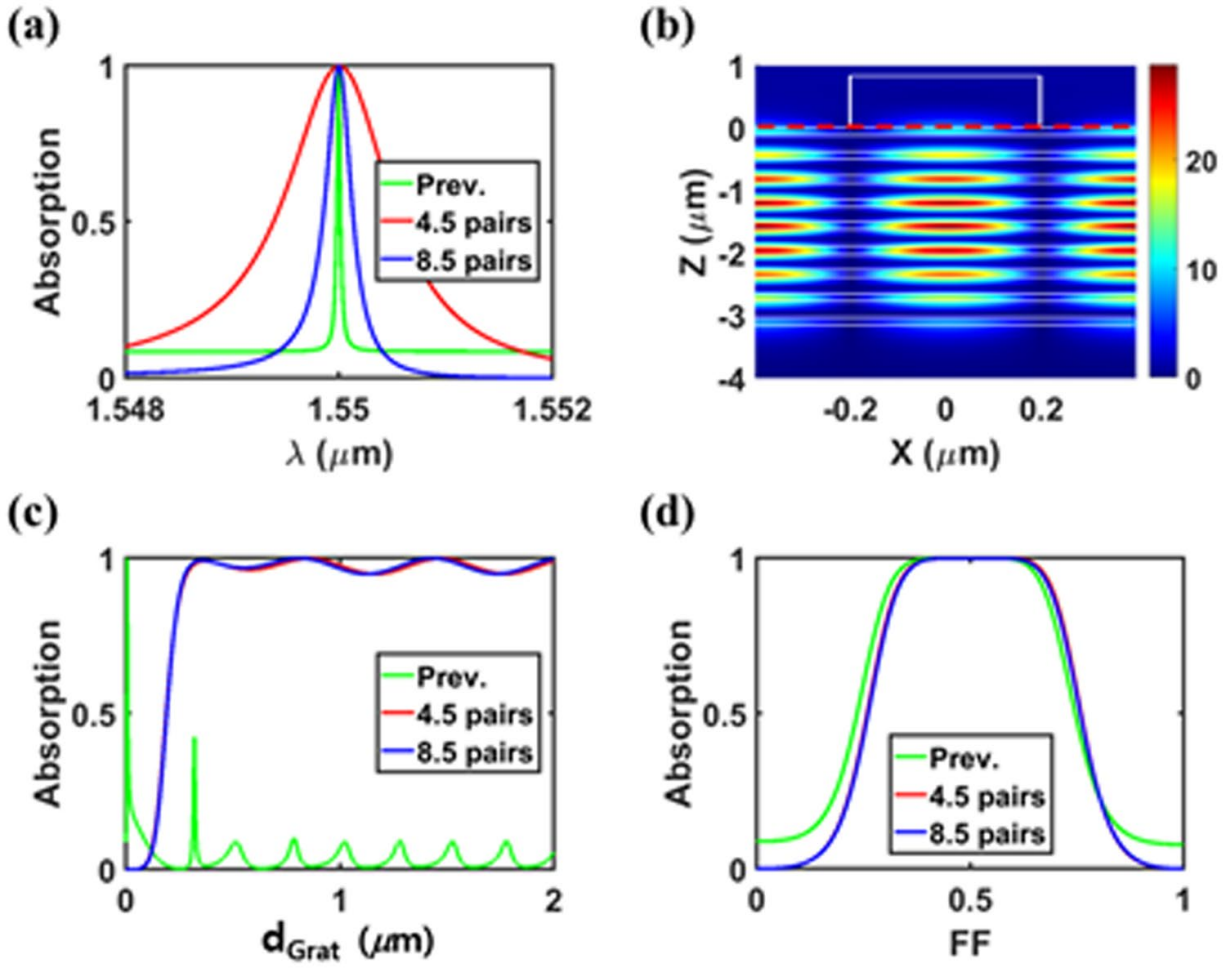
Absorption properties for the proposed absorbers with 4.5, 8.5 DBR pairs, and the previously suggested absorber with 5 DBR pairs. (**a**) Absorption spectra (**b**) Electric field distributions (|*E*_*y*_|) at perfect absorption condition (*A* > 99.9% at *Period* = 0.79764 μm, *FF* = 0.50, *d*_*Grat*_ = 0.82 μm, *λ* = 1.55 μm) for the proposed absorber with 8.5 DBR pairs. Absorption as a function of (**c**) *d*_*Grat*_ and (**d**) *FF*. All the calculations are conducted by assuming the remaining parameters are same as each optimal condition.

The resonant perfect absorber with a narrower bandwidth is very useful for applications such as optical filters, nonlinear optics, and biosensors^22,23^. As seen in Fig. 5(a), the previously suggested HCG-based absorber of 5-pair DBR (FWHM = 0.04 nm) provides the much narrower absorption spectra compared to our proposed absorber with 4.5 DBR pairs (FWHM = 1.2 nm). Although the HCG-based scheme is advantageous for highly wavelength sensitive applications, its practical fabricate is extremely challenging owing to significantly poor tolerance. In our proposed absorber, fortunately, the bandwidth can be reduced to sub-nanometer by increasing the number of alternating-layer pairs in DBR without degrading fabrication tolerance. For 8.5 pairs DBR, the optimized structure of *Period* = 0.79764 μm, *d*_*Grat*_ = 0.82 μm, and *FF* = 0.5 provides a FWHM of 0.3 nm. This is attributed to a decrease of a loss rate due to an increase of the distance between the guided mode and the graphene as seen in Fig. 5(b) where the center of electric fields moves downward compared to Fig. 2(d). Note that the distance between the grating and the guided mode increases simultaneously, resulting in a decrease of a leakage rate. Consequently, the critical condition can be satisfied with slight grating geometry modification. Figure 5(c,d) show absorption at the target resonance wavelength for our designed absorbers of 4.5- and 8.5-pair DBRs as a function of *d*_*Grat*_ and *FF* assuming remaining parameters fixed at the optimal values in each case. For comparison, the absorption of the HCG-based absorber of 5-pair DBR designed in the above section (denoted as ‘Prev.’) is also plotted. One can see high fabrication tolerance is hardly affected by the number of the alternating-layer pairs in DBR for our proposed absorbers. The small absorption variation with a grating thickness change seems to be the effect of weak *FP* resonance in the vertical direction. Note that the small index difference between air and the grating weakens the *FP* resonance effect in our proposed absorber.

## Discussion

In this work, an excellent fabrication tolerance of the grating has been investigated for the proposed perfect absorber of LCG/graphene/DBR configuration. In our design of perfect absorbers, the DBR was assumed to be composed of alternating-index layers of quarter-wavelength for the convenience of simple design. However, similar performance can be also obtained as long as the DBR guarantees high reflectivity, even for DBR having the alternating-index layers of different optical thicknesses. The optimal period of the grating shall be changed correspondingly because the effective refractive index of the guided mode in DBR changes.

In our proposed absorber, the monolayer graphene can be also placed just below the DBR (instead of above the DBR). Since the electric field profile is almost symmetric with respect to center of DBR as seen in Figs 2(d) and 5(b), the monolayer graphene placed just below or above the DBR experiences almost the same loss. The change of the distance between the guided mode and the grating is also negligible when the graphene layer is relocated, resulting in almost the same leakage rate. Consequently, the performance of our proposed perfect absorber and its high fabrication tolerance is still sustained even for the graphene location at the bottom of the DBR with the structural parameters unchanged. (Supplementary Information).

When we fabricate our proposed absorber, graphene can be doped unintentionally depending on the upper or lower adjacent layer material. If graphene is doped, the loss can significantly decrease. According to Kubo formulation, for *E*_*f*_ = 0.5 eV and mobility of 0.5 m^2^/Vs, the loss corresponds to ~1/40 of the undoped graphene case at *λ* = 1.55 μm^20,21^. In this low loss case, it is difficult to find the critical coupling condition for A > 99.9% (that is, ~0.94 < *γ*_leak_/*γ*_loss_ < ~1.06) in the vicinity of *FF* = 0.5 in our proposed absorber scheme because lowing of *γ*_leak_ by increasing DBR pairs is limited. This problem can be solved by adding the gap layer between the LCG and the graphene layer. The leakage rate can be lowered by increasing the gap layer thickness. Thus, by adjusting the gap thickness, nearly perfect absorption (A > 99.9%) can be obtained for *FF* ≈ 0.5 maintaining high fabrication tolerance. (Supplementary Information).

In this work, we have focused on the device performance dependence on the grating shape variation which is effectively quantified by the grating fill-factor and the grating thickness. This is based on the assumption that fabrication error occurs mainly because of imperfect vertical shape control in a patterning or etching process, while the grating period and the DBR layer thickness can be controlled more accurately. Nonetheless, it is meaningful to consider the performance dependence on the period and the DBR layer thickness. So, we conducted the investigation on this issue and discussed in detail in Supplementary Information (section 5). Here, the result is summarized in Table 1 along with the grating shape tolerance. Since all the schemes considered in this work are based on the GMR, the resonance (peak absorption) wavelength is related with the period as 2π/*Period* = *n*_eff_(2π/*λ*) (the phase matching condition), where *n*_eff_ is the effective index of the guided mode, resulting in about the same values of ∆*λ*/∆*Period* in all schemes. For the resonance wavelength dependence on the DBR dimension (∆*λ*/∆*d*_*Pair*_), where *d*_*Pair*_ is the thickness of the single alternating-layer pair in DBR, the previously suggested ‘*Grating-guiding*’ scheme shows the lower dependence, while the other two *‘DBR-guiding’* schemes show about the same dependence. This is because in the *‘DBR-guiding’* schemes, the effective index of the guided mode (*n*_eff_) is directly related to *d*_*Pair*_ and so is the resonance wavelength through the phase matching condition. In spite of the resonance wavelength variation, the absorption bandwidth (FWHM) is hardly affected by the change of *Period* and *d*_*Pair*_. (Supplementary Information).

**Table 1:** Comparison of design parameter dependences (∆*FF*, ∆*d*_Grat_, ∆*λ*/∆*Period*, and ∆*λ*/∆*d*_*pair*_) for the proposed absorber based on *DBR-guiding* scheme, the previously suggested absorbers based on *Grating-guiding* scheme and *DBR-guiding* scheme.

	Proposed (DBR-guiding)	Previous (Grating-guiding)	Previous (DBR-guiding)
∆FF for A > 99%	0.25	0.0015	0.25
∆d_Grat_ (nm) for A > 99%	180	0.26	0.44
∆λ/∆Period	0.9804	0.8333	0.9891
∆λ/∆d_Pair_	2.0494	0.8744	2.0374

In our study, the normal indent wave has been considered like most of the studies on the GMR-based perfect absorber. For an oblique incidence angle, the fabrication tolerance of our proposed absorber degrades to some extent. This is mainly because the scattering property of the grating is incidence angle dependent, resulting in variation of the leakage rate. Whereas, the loss rate of our device is almost independent of the incidence angle because it is mainly determined by the field confinement in the graphene layer which associated with the guided mode profile. As a result, the approximate satisfaction of the critical coupling condition over a wide range of *FF* as shown in Fig. 2(f) deteriorates inevitably. According to our investigation, the deteriorated critical coupling behavior can be restored considerably by slightly modifying the device design. For example, our absorber of *n*_*Grat*_* = *1.70, *Period* = 0.8009 μm, *FF* = 0.506, and *d*_*Grat*_ = 0.39 μm shows about an order of magnitude improved fabrication tolerance compared to the previously suggested structures even for the incidence angle of 10 degrees. So, we believe that high fabrication-tolerant absorber design for a wide incident angle may be possible by modifying our scheme with deeper understanding of the scattering behavior of the grating, which seems to be a rich topic of our future research.

In this work, we only considered a 1D grating for the convenience of simple design and thus, only the TE polarized incident light was assumed. It is straightforward that polarization independent operation is possible if a 2D grating is adopted.

## Methods

To numerically investigate and analyze the absorption properties in the proposed perfect absorber, we used two-dimensional RCWA (a commercial software, DiffractMOD)^17^ and the coupled mode theory (CMT) fitting for a lossy one-port resonant system^5,18^. In the RCWA calculation, more than 300 harmonics were applied to guarantee accuracy near the resonant frequency. For the perfect absorber design, the PSO^19^ was used. In all our calculations, the complex permittivity of graphene (*ε*_*g*_) was calculated using Kubo formulation based on the local random phase approximation for various *E*_*f*_^20,21^, assuming graphene thickness of 0.34 nm, Fermi velocity of 10^6^ m/s, and mobility of 0.5 m^2^/Vs. (Supplementary information).

## Supplementary information

Supplementary information
