# Supplementary material for: High fabrication-tolerant narrowband perfect graphene absorber based on guided-mode resonance in distributed Bragg reflector
Source: Sci Rep. 2019 Mar 12;9:4294. doi: 10.1038/s41598-019-40945-4 (PMC6414689; doi:10.1038/s41598-019-40945-4)
Supplement: Supplementary file 1 — Supplementary information [file 41598_2019_40945_MOESM1_ESM.pdf]

< Supplementary Information >

Title: "High fabrication-tolerant narrowband perfect graphene absorber based on guided-mode resonance in distributed Bragg reflector"

Sangjun Lee, Hyungjun Heo & Sangin Kim*

*Corresponding Author: E-mail:sangin@ajou.ac.kr

Department of Electrical and Computer Engineering, Ajou University, Suwon, South Korea

1. Optical constants of graphene

Optical properties of graphene can be described with a complex permittivity, and its imaginary part is directly related to the loss rate in graphene. In Fig. S1, a real part of a conductivity (σ_G) and an imaginary part of the permittivity (ε_G) of graphene are plotted, which are calculated from Kubo formula for two Fermi-levels (E_f = 0, 0.5eV) with mobility Mo = 0.5m^2/Vs. A higher E_f induces a decrease in loss rate when E_f is sufficiently high so that the graphene becomes metal-like because intraband transition is dominant. For example, for E_f = 0.5eV and λ = 1.55μm, the loss rates decrease to ~1/40 of the undoped graphene's value.

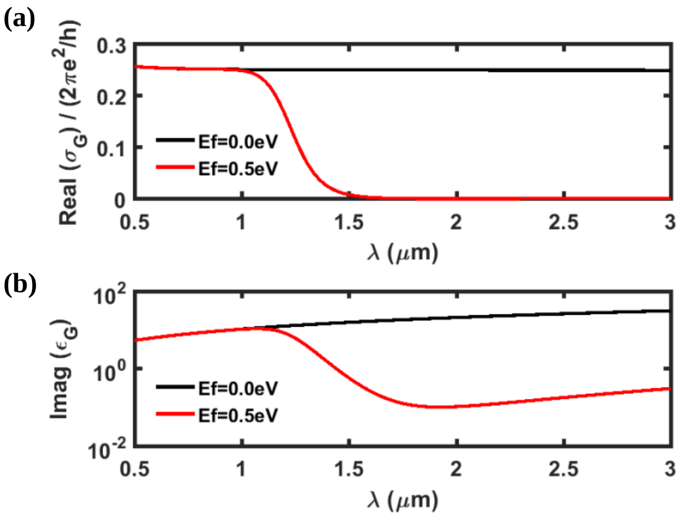

$$\varepsilon_G = 1 + j\frac{\lambda}{2\pi c}\cdot\frac{\sigma_G}{\varepsilon_o t_G}$$

Figure S1. (a) Real part of conductivity and (b) Imaginary part of permittivity of graphene for E_f = 0eV and E_f = 0.5eV. Note that (b) is plotted on a log scale.

2. *DBR-guiding* scheme in previously suggested absorber with thick HCG

As discussed in the main article, we conducted optimization to obtain perfect absorption at λ = 1.55μm when *FF* = 0.50 and 5 DBR pairs is assumed in the previously suggested absorber based on *DBR-guiding* scheme. The perfect absorption occurs at d_{Grat} = 0.00795μm and *Period* = 0.78151μm. Figure S2(a) shows absorption spectra as a function of d_{Grat} for optimal *Period* = 0.78151μm. (Note that Fig. 4(a) corresponds to close-up view of Fig. S2(a) at the thin d_{Grat} ranges). Overall, remarkable distortion of absorption peak branches occurs around λ = 1.55μm, which means strong interaction between *DBR-guiding* and *FP* resonance. The change in resonance wavelength is an obvious evidence of low fabrication tolerance. Figure S2(b) indicates the strong coupling between the two types of resonances, where a comparable field confinement in both regions of DBR and HCG implies that resonant condition is inherently determined by HCG geometry (*FF*, d_{Grat}) and DBR geometry (*Period*). When absorption peak point is far from λ = 1.55μm, almost FP resonance effect is remained, as estimated Fig. S2(c). As a result, if the HCG is thick enough, fabrication tolerance is very poor, as shown in Fig. S2(d). Compared to the LCG of our proposed absorber, due to high index of the grating, the HCG reinforces the *FP* resonance and the change in resonance condition, so that the absorption is significantly affected by the grating geometry. Even if the graphene is placed just below the HCG, the fabrication tolerance is still poor because the strong *FP* resonance occurs regardless of the graphene layer.

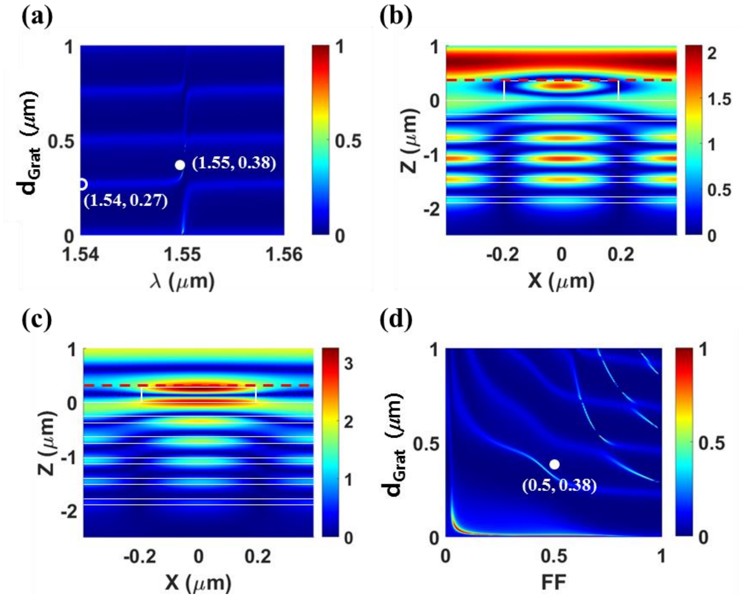

Figure S2. The previously suggested absorber based on *DBR-guiding* scheme (with optimal *Period* = 0.78151μm for 5 DBR pairs). (a) Absorption spectra as a function of d_{Grat} when *FF* = 0.50. Electric field distributions ($|E_y|$) at (b) λ = 1.55 μm, d_{Grat} = 0.38 μm, and (c) λ = 1.54 μm, d_{Grat} = 0.27 μm. (d) Absorption map as a function of (*FF*, d_{Grat}) at λ = 1.55 μm.

3. When the graphene is placed below the DBR

In the proposed absorber, as shown in Fig. S3(a), the monolayer graphene can be also placed just below the DBR. When the graphene position is changed, the loss rate in the graphene is supposed to be changed due to different electric field intensity inside the graphene, while the proposed absorber under the phase matching condition can excite the guided mode with unchanged leakage rate regardless of the graphene. Interestingly, the electric field profile based on *DBR-guiding* scheme is almost symmetric with respect to center of DBR, as checked in Fig. S3(c) or Fig. 2(d). This means that the monolayer graphene placed just below or above the DBR experiences the almost same loss rates. As a result, the trend of high fabrication tolerance with high absorption is still sustained (Fig. S3(b)), assuming that the remaining parameters are the same as those in Fig. 2(c).

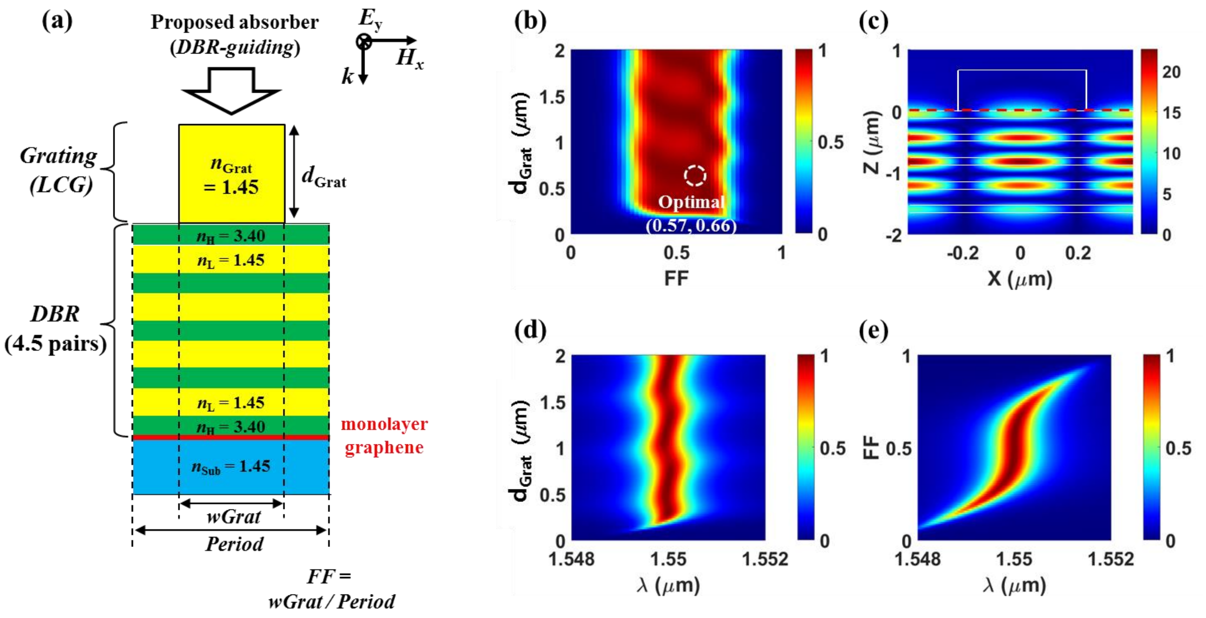

Figure S3. (a) Schematic of the proposed absorber (with *Period* = 0.78344 μm for 4.5 DBR pairs and λ = 1.55 μm) when the monolayer graphene is placed just below the DBR. (b) Absorption map as a function of (FF, d_{Grat}), and (c) electric field distributions ($|E_y|$) at perfect absorption condition (A > 99.9% at FF = 0.57, d_{Grat} = 0.66 μm). (d) Absorption spectra as a function of d_{Grat} when FF = 0.57. (e) Absorption spectra as a function of FF when d_{Grat} = 0.66 μm. In all the RCWA calculations, E_f = 0 eV, Mo = 0.5 m^2/Vs are assumed.

4. Perfect absorber with the highly doped graphene

We can consider the doping effect of the graphene. As previously noted, the loss rate of the guided mode significantly decreases due to the lower loss based on intraband transition in graphene when E_f is sufficiently high. We focus on the graphene with E_f = 0.5 eV at λ = 1.55μm. For the absorber scheme of Fig. 1(a), it is actually difficult to find the condition for A > 99.9% in the vicinity of *FF* = 0.50, where $\gamma_{\text{leak}} \gg \gamma_{\text{loss}}$. This problem can be solved by adding the gap layer between LCG and DBR, as shown in Figure S4. The thicker gap layer induces the smaller leakage rate because the guided mode becomes far from the scattering source. Thus, by adjusting the gap thickness so as to be optimized at *FF* ≈ 0.50, nearly a perfect absorption can be obtained over a wide range of grating size. In our optimal design, d_{Gap} = 0.31 μm, *Period* = 0.781508 μm. When the optimal d_{Grat} = 0.22μm, A > 99.9% at 0.383 < *FF* < 0.528 (Δ*FF* < 0.145). Also, when the optimal *FF* = 0.50, A > 99.9% at 0.203μm < d_{Grat} < 0.263μm (Δd_{Grat} < 60 nm).

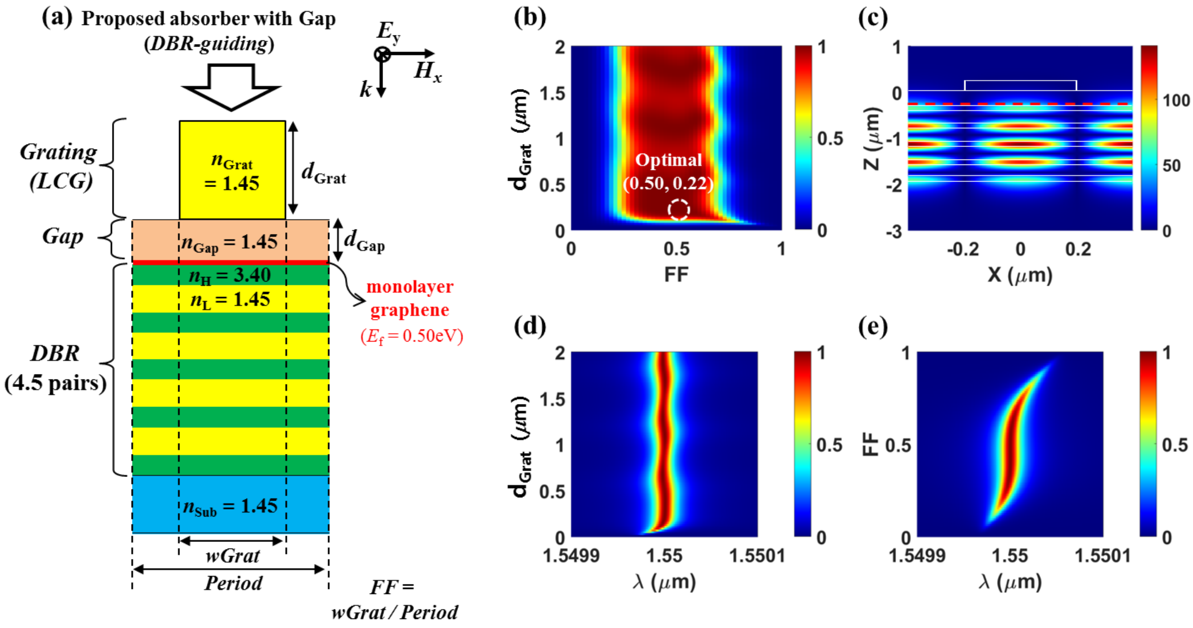

Figure S4. (a) Schematic of the proposed absorber (with optimized *Period* = 0.781508 μm for 4.5 DBR pairs and λ = 1.55 μm) when the highly doped graphene (E_f = 0.5 eV) is applied and a gap layer is added between LCG and DBR. (b) Absorption map as a function of (*FF*, d_{Grat}) for d_{Gap} = 0.31 μm, and (c) electric field distributions ($|E_y|$) at perfect absorption condition (*A* > 99.9% at *FF* = 0.50, d_{Grat} = 0.22 μm). (d) Absorption spectra as a function of d_{Grat} when *FF* = 0.50. (e) Absorption spectra as a function of *FF* when d_{Grat} = 0.22 μm. In all the RCWA calculations, E_f = 0.5 eV, *Mo* = 0.5 m^2/Vs are assumed.

5. Dependency on period and DBR thickness

For three optimized absorbers which discussed in the main article (that is, the proposed absorber based on *DBR-guiding* scheme, the previously suggested absorbers based on *Grating-guiding* scheme and *DBR-guiding* scheme), the dependency on *Period* and d_{Pair} is investigated (Figure S5). d_{Pair} is defined as thickness of unit-pair in DBR. For example, d_{Pair} = 1.55 μm / 3.4 / 4 + 1.55 μm / 1.45 / 4 = 0.38121 μm if the DBR is composed of alternating-index layers of quarter-wavelength at λ = 1.55 μm. As estimated from the phase matching condition of the GMR, 2π / *Period* = n_{eff} x 2π / λ, where n_{eff} is the effective refractive index of the guided mode and depends on DBR thickness, the absorption peak wavelength (or resonance wavelength) strongly depends on *Period* and d_{Pair} for all the absorbers. We extracted the slope from the absorption spectra: $\Delta\lambda$ / Δ*Period* = 0.9804, 0.8333, 0.9891, and $\Delta\lambda$ / Δd_{Pair} = 2.0494, 0.8744, 2.0374 for each absorber. In particular, two *DBR-guiding* based absorbers have considerably higher $\Delta\lambda$ / Δd_{Pair} than *Grating-guiding* based absorber because the main parameter that determines n_{eff} is DBR configuration for the former cases, as estimated in Fig. 2(d) and Fig. 4(d). The resonance bandwidth (or FWHM) is not almost affected when *Period* and d_{Pair} are changed.

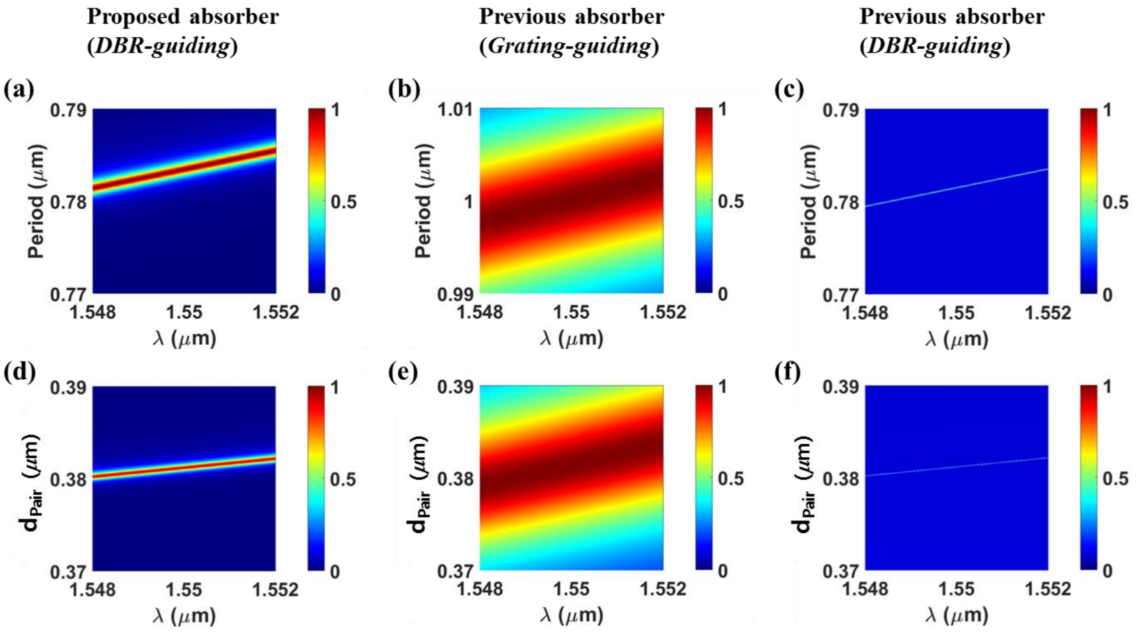

Figure S5. Absorption spectra as a function of *Period* (top panel) and d_{Pair} (bottom panel) for the proposed absorber based on *DBR-guiding* scheme, the previously suggested absorbers based on *Grating-guiding* scheme and *DBR-guiding* scheme. All the calculations are conducted by assuming the remaining parameters are same as each optimal condition which discussed in the main article (Figure 1-3). And, E_f = 0 eV, *Mo* = 0.5 m^2/Vs are assumed.
